# Supplementary material for: Factors associated with willingness to take Pre-Exposure Prophylaxis (PrEP) among high-risk adolescent boys and young men in Masese fishing community, Uganda
Source: PLOS Glob Public Health. 2023 Jun 8;3(6):e0001191. doi: 10.1371/journal.pgph.0001191 (PMC10249897; doi:10.1371/journal.pgph.0001191)
Supplement: Attachment — Submitted filename: Response to Reviewers.doc [file pgph.0001191.s002.doc]

Winnie Agwang,

C/O Makerere University, School of Public Health,

New Mulago Hill Road, Mulago,

Kampala, Uganda.

February 23, 2023

The Editor,

PloS Global Public Health Journal

**Re: Submission of Revised Manuscript titled “Factors associated with willingness to take Pre-Exposure Prophylaxis (PrEP) among high-risk adolescent boys and young men in Masese Fishing Community, Uganda”**

We are pleased to submit our manuscript titled, “Factors associated with willingness to take Pre-Exposure Prophylaxis (PrEP) among high-risk adolescent boys and young men in Masese Fishing Community, Uganda” for publication in PLoS Global Public Health after addressing the reviewer comments raised. We believe the paper is now improved based on these comments. We look forward to your acceptance of this paper. Attached is the response to the comments.

Yours sincerely,

WINNIE AGWANG

Primary and Corresponding author

winnieotuba@gmail.com

+256778192948

Table addressing comments raised by the reviewers.

| **SN** | **REVIEWER’S COMMENT OR SUGGESTION** | **RESPONSE** | **LOCATION** |
| --- | --- | --- | --- |
| **REVIEWER 1: Major comments** | | | |
|  | **Introduction** |  |  |
| 1. | In the first paragraph authors mention that PrEP has been effective in prevention of HIV in high-risk populations, it would be helpful to the readers to quantify how effective it is by citing studies which have been done around Uganda or East Africa if any. | Thank you very much for raising this comment. This has been addressed. The statistics have been included as well as one of the studies, the Partners’ PrEP study which was conducted in Uganda and Kenya, | Page 3, Lines 68-73 of the tracked revised Manuscript |
| 2. | In the second paragraph, the authors report willingness to use PrEP in high-risk populations and MSM in China. It would be important to cite this willingness among the studied population (fishing community) and also cite studies near Uganda to bring similarities in location, culture and HIV risk. | A study done by Ssuna among fisher folk in Uganda has been cited. | Page 3, Lines 79-80 of the tracked revised Manuscript |
| 3. | In the last paragraph, the authors mention that understanding willingness to take PrEP among adolescent boys and young men in a fishing community will provide essential pre-intervention information that can guide the design and implementation of PrEP interventions for ABYM in other fishing communities. Which is a good point, however, this needs more clarity. The readers would benefit to understand why the authors chose ABYM in the entire population, what is special about them compared to the general population? | ABYM were chosen because when we were doing another research, during informal interaction, we found that this group engaged in risky sexual behaviour and yet they have poor health seeking habits which puts them at high risk of HIV given that sex is a booming business at the landing site. ABYM have also been left out in many HIV prevention studies and yet they contribute to new HIV infections. | Page 5, Lines 117-118 of the tracked revised Manuscript |
|  | **Methods** |  |  |
| 4. | In the study setting (first paragraph), the authors did a good job of mentioning the population of the area and prevalence of HIV. Is this prevalence for Masese or fishing communities in general? It would be important to the readers to know why the authors went for Masese and what proportion of the population are ABYM? | Thank you for the compliment. The mentioned prevalence is for fishing communities in general. The authors chose Masese because the landing site has a booming sex business and poor health services and this exposes them to HIV. | Page 6, Lines 149-150 of the tracked revised Manuscript |
| 5. | In the second paragraph the authors describe how they calculated the sample size of the study. However, they did not provide the readers with the power calculations of the Poisson regression which was used for associated factors. | We did not include the power calculation because the sample size was large enough and therefore had enough statistical power. |  |
| 6. | In the third paragraph, the authors describe convenience sampling as a method of sampling participants used. Why did the authors decide to use a non-probability sampling method in such a community over other methods like systematic sampling? Wouldn’t this lead to selection bias for only those who were willing to convene in the suggested place? | This is true that convenience sampling can lead to bias. We however used it because because of the varying schedules of the participants making accessibility difficult where some fish at night and some during the day. The fishing communities are also characterized my high mobility and so other sampling methods may give a high non-response rate and for Masese in particular, there is no clear record of the ABYM in the community. We also mentioned it as a limitation and how we tried to reduce on its effect | Page 7, Lines 167-170 of the tracked revised Manuscript.  Page 22, Lines 421- 422 of the tracked revised Manuscript (For the limitation) |
| 7. | In the section of measurement of variables, the authors said the primary outcome was willingness to use pre-exposure prophylaxis (PrEP) which was defined as a participant responding with ‘likely’ or ‘very likely’. However, this did not create a clear-cut difference in those who are likely to use PrEP and whose who were unlikely to use PrEP since both answers are positive. The readers would benefit from explaining to them how the negative responses of those who don’t want PrEP or unlikely to use PrEP was achieved. | More explanation has been given to give more clarification on how the difference came about. It was a single question with the answers on a 5-point Likert scale. Those who responded with very likely and likely were considered willing while those who responded with not sure, unlikely and very unlikely were considered not willing. | Page 8, Lines 183- 189 of the tracked revised Manuscript |
|  | **Results** |  |  |
| 8. | In Table 2, how did the authors differentiate between “very high risk” and “at risk” while asking the participants? | The participants were asked for their self-perceived risk of HIV acquisition. The responses were on a 4-point Likert scale- Very high risk, At risk, not sure and Not at risk. | Page 8, Lines 194- 199 of the tracked revised Manuscript |
| 9. | In Table 3 and 4, the authors reported a single proportion of willingness to use PrEP. The readers would benefit to know the two sides of willingness to use PrEP since it was measured as “likely” and “very likely”? Who were willing and unwilling to use PrEP? | We took note of this. Thank you very much. This has been included on table 3. For table 4, only those who were deemed willing were further asked about their willingness basing on the different PrEP attributes. | Page 15 of the tracked revised Manuscript |
| 10. | In Table 4, how did the authors differentiate between “likely” and “unlikely” responses and the outcome of willingness to use PrEP which was measured as “likely” and “very likely”? | These were only administered to those deemed willing. Forexample for cost, we asked, “How likely are you to take PrEP even if you had to pay for it?” | Page 8, Lines 201- 208 of the tracked revised Manuscript |
|  | **Limitations** |  |  |
| 11. | The study did not intend to find out causal effect, therefore it cannot be a limitation as mentioned in the first sentence | Thank you for the comment. This has been removed though we felt highlighting it would give some clarification to the readers so that they don’t confuse causality with association. |  |
| 12. | One limitation would be the gender role which may play an influence of men to use PrEP which was no captured in this study | This has been noted and we have included it in the limitations. | Page 22, Line 418 of the tracked revised Manuscript |
|  | **Minor comments** |  |  |
|  | **Introduction** |  |  |
| 13. | In the last sentence of paragraph one the authors introduce a concept of adherence are reported that it is directly proportional to willingness to take PrEP. The study which was cited is a randomized trial which doesn’t bring out the picture | This study was added because the findings showed that the prophylactic effect was directly proportional to the blood drug levels. For someone to achieve prophylactic drug level, they must have adhered well to their medication. We have also added more information from the CDC. | Page 3, Lines 75- 76 of the tracked revised Manuscript |
| 14. | In the last sentence of paragraph two, the authors mention about increase in men’s risk of HIV transmission. This sentence needs more specificity, the readers would benefit from more information for example what is the transmission rate compared to Uganda general population? | Thank you for the comment. Transmission rate comparison would have been good but unfortunately, we did not find any data on that. We there fore gave the statistics on prevalence both in the fishing communities and among the men in the two groups. | Page 4, Lines 108- 109 of the tracked revised Manuscript |
|  | **Methods** |  |  |
| 15. | In the section of ethics, the authors would explain more on how they assented the minors. From the sampling technique used, did the minors come with their guardians at the place of convince? And how was confidentiality achieved? | This has been expanded further for purposes of clarity. The research assistants went with the minors to their homes to seek consent from the parents and for some we asked them to come with the Parent to the place of convenience to give their consent. Privacy and confidentiality were also observed during data collection by use of participant numbers and carrying out the interview away from other participants respectively. The participants were also informed that they could leave the study whenever they wanted. | Page 11, Lines 255- 260 of the tracked revised Manuscript |
|  | **Conclusions and recommendations** |  |  |
| 16. | In the last sentence, the authors mention this shows that adolescent boys and young men in fishing communities are a suitable population for HIV prevention. However, this study was not aimed at determining suitability of the population. Readers would benefit in conclusions and recommendations around the study aim. | Thank you very much. We have taken note of this and deleted what is not around the study aim. | Page 22, Lines 435- 438 of the tracked revised Manuscript |
| **REVIEWER 2** | | | |
|  | The manuscript addresses an important topic among high risk young men who are often times not the focus of research yet interventions such as oral PrEP are beneficial to this group and their sexual partners. | Thank you very much for the kind comments. We appreciate it. |  |
|  | **INTRODUCTION** |  |  |
| 1. | Lines 71-72 give some background data on willingness among men outside Africa. Do also compare with studied in Africa or other young people e.g., adolescent girls and young women (AGYW). This study doi:10.1002/jia2.25909 (done among AGYW in Kampala has results on willingness to take oral PrEP even though its focus was mainly uptake and adherence. | Thank you very much for the guidance. Unfortunately, we could not access this study. However, a study done by Ssuna among fisher folk in Uganda has been cited as well as those done by Muwonge and Ddaaki who did studies among high-risk populations which included fisherfolk. We also included a study by Muhumuza which looked at young people. | Page 3, Lines 79-80 and Page 4, Lines 88-94 of the tracked revised Manuscript |
| 2. | Line 78 mentions different populations, yet studies referenced in lines 73-78 are mainly among MSM with one done among opium users. Qualify this statement by highlighting which willingness factors are unique to MSM, drug users, fisherfolk etc and which factors are commonly found in most high-risk male populations (would also be the same factors for females). You can also read the literature to find if there are differences between younger men like ABYM and older men. | We revised this and grouped the factors associated with willingness based on the different populations. | Page 3-4, Lines 81-94 of the tracked revised Manuscript |
| 3. | Lines 83-85, fisherfolk also have disposable income so usually go for sex workers since they can afford to pay. | Thank you for the suggestion. This has been added to the manuscript | Page 4, Lines 105-106 of the tracked revised Manuscript |
| 4. | Lines 86-88; Keep consistency in the decimal place for results written in percentages. | This was noted and applied throughout the document. |  |
| 5. | The gap is not well articulated at the end of the introduction. Before the authors mention “understanding willingness”, they should mention what the gap in literature is that is driving the research question. This is related to point 1 above, if the authors have searched the literature on willingness among ABYM and it is lacking, do mention it. | ABYM were chosen because when we were doing another research, during informal interaction, we found that this group engaged in risky sexual behaviour and yet they have poor health seeking habits which puts them at high risk of HIV given that sex is a booming business at the landing site. This however, we could not support with literature although it is also true that ABYM have been left out in many HIV prevention studies and yet they contribute to new HIV infections. So, we wrote the gap around those two areas. | Page 5, Lines 117-118 of the tracked revised Manuscript |
|  | Methods |  |  |
| 6. | Line 110, The title could include sampling methods rather than sample size. Details of the sample size calculation are better suited for the study protocol but sampling methods used in the study are important to include here. | This has been included in the title. Thank you very much for the suggestion. | Page 6, Line 143 of the tracked revised Manuscript |
| 7. | Lines 118-119; This statement does not describe the study setting but rather a general statement. “However, PrEP in fishing communities is 119 not yet fully embraced despite having HIV prevalence as high as 37%” It could be why you want to study willingness so can be taken to the introduction | We thought it would be good for the readers to get to know about Masese. The statement feels general because most fishing communities are more or less the same and Masese is no exception.  The last part of this paragraph has been taken to the introduction. | Page 6, Lines 146-150 of the tracked revised Manuscript  Page 5, Lines 114-117 of the tracked revised Manuscript |
| 8. | The sample of ABYM include minors, how did the VHTs and other ABYM reach these boys without their parents/ guardians involved. Briefly clarify what the process was for those who were <18 years. This is mentioned in ethics section but also needs a brief here on emancipated minors, parental consent etc | Thank you for the suggestion. We however feel like it will be a repetition and so we decided to leave it in the ethics but expand it a little more. | Page 11, Lines 257-258 of the tracked revised Manuscript |
| 9. | Line 138, qualify the partner’s status by specifying “HIV” | This has been added to qualifu the partner’s HIV status. | Page 7, Line 178 of the tracked revised Manuscript |
| 10. | Lines 158-162 repeat information already given in the section before on study variables and, also given below in the “measurement of variables”. Re-do or merge some of these sections to avoid repetition. | Thank you for the suggestion. These sections have been merged to avoid repetition. | Page 8, Lines 183-233 of the tracked revised Manuscript |
| 11. | Line 169; give the whole range of possible responses on the measurement scale to give readers a clue of what the responses were for participants classified as “unwilling” | We have edited the manuscript to give the whole range of responses. | Page 8, Lines 183-189 of the tracked revised Manuscript |
| 12. | Line 171, what was the cut-off or categories to define perceived risk and no-perceived risk? Please mention this for clarity so that it is understood when the reader goes to the results section. | We have also edited to add this. | Page 8, Lines 194-199 of the tracked revised Manuscript |
| 13. | Study variables: mention whether the STI diagnosis was syndromic approach or laboratory tests were done. If labs were done, describe the assay under study procedures. Ensure that it is clear how you measured all the other variables. | The STI diagnosis was a self-report by the participants and so it included both syndromic and lab tests. We have however included it for clarity purposes. | Page 8, Lines 193-194 of the tracked revised Manuscript |
| 14. | Line 178; was there any double entry and data cleaning? Single entry in EpiData may have errors that go unnoticed if no active cleaning is done. | Yes, there was double data entry. We just felt that that would be too much detail. However for purposes of clarity, we have included it in the revision. | Page 10, Lines 238-240 of the tracked revised Manuscript |
| 15. | Line 179, add “if normally distributed” after means otherwise it looks like “if they were skewed” applies to both the means and medians. | Yes, this is true. Thank you for the suggestion. This has been added for clarity purposes. | Page 10, Lines 240-241 of the tracked revised Manuscript |
| 16 | Lines 187-188: Check the last sentence, it doesn’t read well/ is incomplete. | Thank you. This has been edited to read well. | Page 11, Line 249 of the tracked revised Manuscript |
| 17. | Lines 190-198; Did this study not require approval by the national council (UNCST)? | No. The study did not require UNCST approval. It was approved by the Research and Ethics committee of Makerere University, School of Public Health. | Page 11, Lines 252-253 of the tracked revised Manuscript |
|  | **RESULTS** |  |  |
| 18. | Lines 224-225; Rephrase this sentence and include the chi square p-value. The results show that a higher proportion of those who perceived themselves at risk of HIV were willing to take PrEP compared to those who had lower risk perception. The results are proportions and don’t show that willingness increased with higher risk perception. | Thank you very much for the suggestion. Unfortunately, we did not use Chi square analysis for the bivariate. We used regression analysis at this stage. So we presented the results as prevalence ratios with their confidence intervals as per the analysis plan. | Page 15, Lines 299- 310 of the tracked revised Manuscript |
|  | **DISCUSSION** |  |  |
| 19. | Line 263; Rather than repeat results, mention if this level of willingness is high/ low/ expected compared to other literature | The repetition has been deleted and comparisons made to other studies, | Page 18, Lines 328- 340 of the tracked revised Manuscript |
| 20. | Line 268 is a repetition of lines 263-264. Delete one. | This has been deleted. Thank you. |  |
| 21. | Lines 268-271; The willingness in your study is much higher than studies you qualify as having results in the same range. Revisit this statement and interpret results correctly in relation to other studies. | Thank you for this observation. This was an oversight on our end but we have corrected it. | Page 18, Lines 334- 335 of the tracked revised Manuscript |
| 22. | Lines 272-273; The results of these 2 studies and yours are in the same range compared to those in lines 268-271. Improve the discussion here. Discuss these studies with high willingness well in relation to yours (especially reference 36). Looks like young boys/ adolescents have high willingness. | This had been discussed according but in relation to the Ssuna study as these were done in similar settings. | Page 18, Lines 340-342 of the tracked revised Manuscript |
| 23. | Line 326; Those earning more money are also more likely to afford condoms and therefore rightly perceived themselves at lower risk which supports their unwillingness since they already use condoms for HIV prevention. Enrich your discussion further using literature. | Thank you for the suggestion. We have incorporated it to enrich the discussion though the literature is really scanty. | Page 21, Lines 397-399 of the tracked revised Manuscript |
| 24. | Lines 3545-346; Look within your data and see if those who had high risk perception and reported high risk behaviors also had STI symptoms. Although STIs are sometimes asymptomatic, corroborating these variables within your data may give a clue to inconsistencies and if desirability bias was really a major issue or it occurred at a lower frequency. | We tried to do this but there were no significant differences found and so we decided to leave as is and go with literature. |  |
|  | **Referencing** |  |  |
|  | Use Vancouver style as in the author guidelines and read instructions to authors to ensure all manuscript sections conform to journal requirements. | Thank you for the suggestions. We have read the author guidelines and the current manuscript revision follows author guidelines. Thank you very much for the guidance. |  |
